# Unobtrusive Observation of Team Learning Attributes in Digital Learning

**DOI:** 10.3389/fpsyg.2018.00834

**Published:** 2018-05-31

**Authors:** David C. Gibson

**Affiliations:** Learning Futures, Curtin University, Perth, WA, Australia

**Keywords:** unobtrusive observation, continuous assessment, digital learning, higher order thinking, learning analytics

## Abstract

This article presents a new framework for unobtrusive observation analytics of knowledge and skills-in-action through continuous collection of data from individuals while they interact with digital assets either as individuals or on problem-solving teams. The framework includes measures of the skill and knowledge areas of collaboration, creativity, personal learning, problem solving, and global sustainability, which are observed during natural production and use of communications, intentional artifacts, and resources in a digital learning space designed for self-directed and team-based learning challenges. The article describes the digital context for data collection and shows some example data and analyses.

## Introduction

Digital learning environments present new opportunities for analytics-based learning design (Shum and Ferguson, 2011; Scheffel et al., 2012; Gibson and Ifenthaler, 2017), assessments (Gibson and Webb, 2013; Griffin and Care, 2015), and learning supports such as scaffolding for self-regulation (Ifenthaler, 2012). In the case of assessments, the new opportunities for analytics can be extended to include legitimate peripheral participation (Lave and Wenger, 1991) in team learning by documenting individual behaviors, actions, and problem-solving strategies while working in the social setting of team work (Kinshuk Ifenthaler et al., 2010). Digital games and simulations designed for team performance for example, often are characterized by integrated, media-rich contexts with multiple layers of interaction with peers as well as computational resources, which provides a foundation for authentic performance of individual and team-based problem-solving processes (Clarke-Midura et al., 2012) with attendant opportunities for unobtrusive observation and documentation of strategies, tools, communications, intentional actions and artifacts (Griffin et al., 2012; Siadaty et al., 2015).

In this article, a foundation is outlined for unobtrusive observation of the knowledge and skills exhibited in an online challenge-based learning platform (Gibson et al., n.d.) during collaboration, problem-solving, personal learning, creative thinking, and working with concepts of global sustainability elicited by real-world challenges. Definitions of key terms and measures of a specific set of team learning attributes are provided and a proposed new integrated mapping is illustrated for sample interactions in an application called ‘Balance of the Planet’ that will serve as a concrete case example. A proposed general method for planning for unobtrusive observations involves metadata mapped to structured and unstructured digital interactions where an individual or group’s intentions are explicitly prompted and play a role similar an assessment prompt. However, in team problem solving addressing complex challenges in a digital learning space, there is considerable openness in the type, degree and amount of possible learner responses documented by a highly granular data record of a learner’s performance, with many attendant options for analysis.

## Team-Based Learning Attributes

As learners utilize resources and performance affordances of a well-designed digital learning space, they touch things, comment via text or talk, upload and download files, listen and watch, and leave other kinds of evidence of their *interactions* in a time-based digital record. If the interactions have been thoughtfully assembled to support a chain of evidence, then a learner’s thinking patterns and actions can be observed and inferences can be drawn concerning what they know and can do, including ‘knowledge of’ and ‘knowledge-in-action’ of valued constructs (Quellmalz et al., 2012; Gibson and Jakl, 2015).

The constructs of ‘collaboration, problem-solving, personal learning, creativity, and global sustainability’ outlined below (**Table 1**) are drawn from multiple sources in the research literature on digital assessment and serve as part of a high-level *domain model* (Mislevy et al., 2003) for the observation of team learning. The domain model helps organize potential claims about learner proficiency; the kinds of things students might say or do that would constitute evidence about these proficiencies; and the kinds of situations that elicit relevant evidence.

**Table 1:** Construct definitions of team learning attributes in Balance of the Planet.

**Collaboration**: coordinated group activity resulting from continuous attempts to construct and maintain a shared conception of a problem (Roschelle and Teasley, 1995).
**Problem solving:** cognitive processing directed at achieving a goal when no solution method is obvious (Mayer and Wittrock, 1996).
**Personal learning**: acquisition of knowledge (e.g., new insights, capacities for thinking, acting and employing skills) that is evidenced for outside observers as well as an individual’s own reflection and metacognition (Friedrichs and Gibson, 2003).
**Creativity**: creative solutions are novel (surprising and original), effective (useful and understandable) and whole (sensitive to the context of their creation) (Mishra et al., 2013).
**Global sustainability**: development that meets the needs of the present without compromising the ability of future generations to meet their own needs (Brundtland, 1987).

Second-level components in the domain model (**Table 2**) define sub-events that are partial measures for each construct. For example, collaboration is evidenced by three activities needed to construct and maintain a shared conception of a challenge: reaching a shared understanding, taking appropriate action to address the challenge, and maintaining group organization. If any of these sub-components fail, then collaboration fails. Thus, the second-level components must all be observed and validated over a body of evidence over time in the digital record in order to enable a confident inference that the attribute has been identified for an individual. Because multiple observations are required over time, trajectories of the attributes are expected, that is, the attributes are expected to be seen more than once and in different contexts, and may be seen to evolve or change in some way during the time span of the multiple observations.

**Table 2:** Second-level components that define the metadata for unobtrusive observations of team-based learning attributes in Balance of the Planet.

Constructs	Second-level components
Collaboration	(C1) Establishing and maintaining shared understanding
	(C2) Taking appropriate action to solve the problem
	(C3) Establishing and maintaining team organization
Creativity	(CR1) Idea generation
	(CR2) Design and refinement
	(CR3) Openness and exploration
	(CR4) Working creatively
	(CR5) Creative production
Personal learning	(PL1) Sharing experience
	(PL2) Examining diverse concepts
	(PL3) Articulating, applying, and building understanding
	(PL4) Communicating new powers and creations
Problem solving	(PS1) Exploring and understanding
	(PS2) Representing and formulating
	(PS3) Planning and executing
	(PS4) Monitoring and reflecting
Global sustainability	(GS1) Recognizing and valuing the needs and cultures of others
	(GS2) Active involvement in addressing global needs
	(GS3) Supporting development of social, economic and environmental pillars

Provisions for individual performance over time within a group setting ensure that the observable has both individual and group characteristics, through what has been termed the ‘social learning capacity’ of the group (Gibson et al., in press).

To triangulate the measures of each of the key terms, second-level components (**Table 2**) have been identified and form the definition of metadata codes that are directly associated with digital interactions. The second-level components of Collaboration and Problem Solving are drawn from the collaborative problem solving framework of the Programme for International Student Assessment (PISA) a worldwide study by the Organisation for Economic Co-operation and Development (PISA, 2013). The components of personal learning are drawn from research on the personalization of learning (Friedrichs and Gibson, 2003; Gibson, 2003). Second-level ideas about creativity are based in the work of the Deep Play Research Group at Arizona State University (Mishra, 2012; Mishra et al., 2013). Finally, the second-level ideas associated with global sustainability are drawn from the concept of the ‘triple bottom line’ in sustainability research (Elkington, 1997).

## Integrated Mapping of Attributes

The context for mapping the attributes to automated data collection and observations differs from mapping the constructs of a typical test or quiz in two important respects: the learner is unaware of being tested, and the learner has the freedom to act, think, and communicate without an additional cognitive load of evaluated meaning or consequences. I’ll refer to these as the *natural production of evidence* and *self-direction* (including team self-direction) in exploratory learning. Outside of these two aspects, the integrated mapping of attributes to interactions proceeds as part of the development of a chain of reasoning from evidence to inferences guided by a domain model (Almond et al., 2002).

In the following section a case example of a digital learning platform designed for self-directed learning by individuals and teams is used to illustrate. The Curtin Challenge platform delivers a team-based learning opportunity called *Balance of the Planet* and serves as a specific case of a generic model of *backward design* thinking (Wiggins and McTighe, 1998; Guskey, 2014) in which a learning designer *begins with the end in mind*, by thinking about what a learner or learning team should be able to exhibit that they know and can do as a critical performance of their *knowledge-in-action* (Argyris, 1993). The designer begins with the end in mind by articulating the key criteria and performance levels for a critical performance, and creates one or more defining moments when students or a team must display and flexibly use what they know in order to accomplish some cornerstone task. A helpful metaphor is a sports team, whose coach is not on the playing field on game day, but who has prepared the team for success and then watches and encourages from the sidelines while the team faces the challenge first-hand. In professional contexts, *cognitive apprenticeships* (Resnick, 1984; Collins et al., 1989) of various kinds prepare people to ‘act as’ the professional when called upon by circumstances; for example, a doctor-to-be might diagnose a disease during hospital rounds, a teacher-to-be might teach a practice lesson, and an engineer-to-be might take part as a legitimate peripheral team member during a field site inspection. In game and simulation-based learning authentic decisions similar to cognitive apprenticeships can be encountered and the feedback provided to the learner can in many cases be automated or built into the immersive experience.

Authoring a digital learning experience on the Challenge platform follows this basic outline. It starts with the end in mind by defining a package of work products and processes (deliverables for individuals or teams). The application platform then automatically generates scaffolding pages, which break-down the parts of the work package into subtasks and focuses on the feedback based on the criteria needed to achieve success on the deliverable. On each page and linked to each interaction are metadata codes (**Table 2**) and embedded hints (see for example “Extended Prompt” and “EvalVotePrompt” in **Table 3**) that are used either in near real time or *post hoc* to group the evidence into clusters for further processing, display and for reflection on learning.

**Table 3:** Fields and content for a subtask in Balance of the Planet ‘Global Business Plan’ contest.

Field name	Example	Explanation
Task	Global Business Plan	Names the medium-term goal that needs to be reached before progressing on the long-term goal of the challenge (e.g., to create a solution to global warming for a scholarship contest). Groups all subtasks into one large task.
Subtask	Title page	Names the immediate goal of this activity page and the purpose of its interactions.
SubTask_Order	1	Sorts this subtask page on a display page.
Artifact	Product, service, or process name	Generic name of the artifact expected on this page.
Artifact_Order	1	Sort order if more than one artifact is to be uploaded.
Type (one of: text | upload_text | upload_video | upload_image | judge_only | date | acknowledgment)	Text	Defines the type of file to be uploaded.
Prompt	What is the name of your product, service, or process?	Prompts the team to name its solution.
Extended prompt (for iframe extended help)	The name has a big role in creating ‘brand awareness’ of your idea. A good brand creates a positive response in people and a desire to want or learn more about the brand.	Provides a hint and additional information about the prompt for a name for the solution.
EvalVotePrompt (display for team scoring)	How well did the team do in following formatting guidelines?	Prompts the team members to reflect on something specific about the uploaded artifact.
Score	1	Provides a value for acceptable performance via the uploaded artifact. Shows the team how much effort to put into this aspect compared to other team deliverables and requirements.
[Meaning of the Outcome Tag] – use in analysis but do not display until Team Scoring	To what extent did you represent and formulate solution options?	Prompts for reflection after this page’s artifact is ready for submission.
Learning outcome tag	ps2	Metatags the primary evidence collected in relationship to this page of activity (e.g., problem solving 2 = representing and formulating).

In addition to these forms of structured data, the Challenge platform integrates the CISCO Spark application to create team spaces on each sub-task page. The Spark tool provides a flexible persistent messaging system that documents who says what, who shares files with whom at what times and in what contexts, how a conversation, document or product evolves and how decisions are reached related to each subtask. The data stream is addressable by future bots that can function as smart team members that can leverage the open APIs of the platform.

To illustrate, **Table 3** displays a sample of the fields and content for a subtask in Balance of the Planet ‘Global Business Plan’ contest. The subtask is the creation of a product name that will appear on the title page of a final report. The artifact is a text and the key prompts and hints for creating the name include the phrases “*What is the name of your product, service or process?*” and if further information is desired “*The name has a big role in creating ’brand awareness’ of your idea. A good brand creates a positive response in people and a desire to want or learn more about the brand*.” The evidence created for later assessment is tagged as an example of the group’s problem-solving ability connected with representing and formulating an idea (PS2 from **Table 2**). The team members will all vote on how well they think the name meets the criteria. At the same time, the team process of talking about which name to settle on, who proposed the name, who proposed any edits, and so forth, are all saved as data and context for the PS2 evidence.

The process of turning session log files and process stream data into indicators has been recently summarized in Griffin and Care (2015) which also notes several precursor research projects and results in digital media learning, so will not be reduced further here except to say that a process of exploratory data analysis is required based on *post hoc* analysis of real people using an appropriately designed digital space to learn. The growing field of learning analytics focused on learning and learners (as opposed to teaching, institutional progress, curriculum, and other outcomes) is exploring and expanding the knowledge base concerning the challenges and solutions of the layered and complex analyses required nowadays for a better understanding of the impact of digitally enhanced learning spaces on how people learn.

Collecting evidence at such a fine-grained level (e.g., one small component of a title page) of a sub-task (e.g., the title page of a report) of a larger task (e.g., a report about an extended group project) is made feasible by automatic data collection and the nearly full digitization of the group’s process. This approach to data collection and tagging supports assessment inferences based on the natural production of evidence during authentic teamwork and is intended to minimize disturbance of learning in the natural evolution of self-motivated team-based learning.

A limitation of any educational measurement system is that a learner’s thought processes are not directly accessible and are not comprehensively represented in the externalized artifacts of interactions (e.g., the words, images, discussion, products created, and resources used) during learning. This results in a need for learning analytics researchers to make inferences about what someone knows and can do based on limited available evidence. The framework and methods outlined here represent a plan to increase the number and quality of data captures of the natural production of artifacts during individual and team problem-solving in order to increase the data resolution (e.g., the fine-grained data details) of the processes of externalizing thought and learning processes.

## Conclusion

A key difference between being tested and unobtrusively observed is the extent to which the production of actions arises naturally without awareness or anxiety about being watched and evaluated. The digital platform can be designed for unobtrusive observation and capturing salient solution and construction processes of a learner’s natural production of evidence via *communicating, making artifacts* for a known purpose (e.g., the subtask in a context), and *using resources* during the processes of acquiring and organizing information, creating responses and things that can be (or for which images can be) digitally uploaded and communicating ideas to others.

A second important construct of a digital challenge-based learning platform is based on the assumption that learners can make their own way toward a production or behavioral goal with minimal assistance, passing through various cognitive states along a trajectory toward a final submission when their goal has been met or their work completed. In addition, as they are making their way, having peers along on the journey raises the stakes of thinking and performance, raises the standards for completion, and provides valuable enhancements to individual knowledge and action.

This article has shared the details of a specific example mapping system for unobtrusive observation of higher order skills (**Table 2**) evidenced during open-ended and self-directed team-based learning on the Curtin Challenge platform. Data now being collected on over 25,000 students is developing the baseline for establishing process stream indicators of the attributes of natural actions involved in learning, problem-solving, and teamwork.

## Author Contributions

The author confirms being the sole contributor of this work and approved it for publication.

## Conflict of Interest Statement

The author declares that the research was conducted in the absence of any commercial or financial relationships that could be construed as a potential conflict of interest.
